# Dietary Habits, Nutritional Knowledge, and Quality of Life Among Patients with Gastrointestinal Cancers: An Exploratory Cross-Sectional Survey

**DOI:** 10.3390/jcm15176508

**Published:** 2026-08-22

**Authors:** Katarzyna Antosik, Damian Dyńka, Elżbieta Krzęcio-Nieczyporuk, Maja Księżopolska, Milena Kobylińska, Katarzyna Kurowska

**Affiliations:** Institute of Health Sciences, Faculty of Medical and Health Sciences, University of Siedlce, 08-110 Siedlce, Poland

**Keywords:** gastrointestinal cancers, quality of life, dietary habits, nutritional knowledge, patients with cancer

## Abstract

**Background**: Cancer is one of the leading causes of morbidity and mortality worldwide, and epidemiological projections indicate that its incidence will continue to increase. Gastrointestinal cancers are among the most frequently diagnosed malignant neoplasms. Quality of life is a key component of oncology care and is significantly influenced by nutritional status and dietary habits. **Methods**: The study included 52 adult patients diagnosed with gastrointestinal cancer. Data were collected using an anonymous questionnaire based on the QEB (Questionnaire of Eating Behaviour) and WHOQOL-BREF (World Health Organization Quality of Life—BREF) instruments, supplemented with questions regarding changes in dietary habits and the use of dietary counseling following diagnosis. Nutritional knowledge, dietary habits, and quality of life across four domains (physical, psychological, social, and environmental) were assessed. Statistical analysis was performed using descriptive statistics and appropriate tests of statistical significance. **Results**: Cancers of the colon and rectum—reported either as unspecified colorectal cancer or as separately specified colon or rectal cancer—together accounted for the largest share of diagnoses (26 diagnoses, representing 44.8% of the 58 total reported diagnoses). Quality-of-life assessment revealed the lowest scores in the physical domain and the highest scores in the social domain. The youngest participants achieved the highest scores in the psychological domain, whereas the lowest scores across all domains were observed among individuals aged over 60 years. Following diagnosis, 63.5% of participants reported reviewing and modifying their dietary habits; however, the majority had not received dietary counseling. Women reported higher fruit and vegetable consumption than men, while higher educational attainment was associated with greater self-assessed nutritional knowledge. **Conclusions**: Among patients with gastrointestinal cancers surveyed in this study, the physical domain of quality of life had the lowest WHOQOL-BREF score. Most participants reported modifying their dietary habits after diagnosis, although most had not received formal dietary counseling. Higher educational attainment was associated with higher self-assessed nutritional knowledge. Given the small convenience sample and cross-sectional design, these findings should be considered exploratory.

## 1. Introduction

Cancer is the second leading cause of death worldwide, following cardiovascular diseases, accounting for nearly one in six deaths globally, according to the latest data from the World Health Organization (WHO) [1]. It is estimated that, in 2026 alone, approximately 2,114,850 new cancer cases and 626,140 cancer-related deaths will occur in the United States [2]. Gastrointestinal malignancies, including colorectal cancer, rank among the most frequently diagnosed cancers worldwide, alongside breast, prostate, and lung cancers, as reported by the WHO [1]. A similar pattern is observed in Europe. According to the European Cancer Information System (ECIS), colorectal cancer was the second most commonly diagnosed malignancy in Europe in 2024, accounting for approximately 12–13% of all newly diagnosed cancer cases and approximately 12.4% of cancer-related deaths [3]. Furthermore, data from the American Cancer Society (ACS) indicate that five of the ten deadliest cancers, based on mortality rates, originate in the gastrointestinal tract [4]. Epidemiological data from Poland indicate that colorectal cancer is the third most frequently diagnosed malignancy in both men (11.7%) and women (8.6%), following prostate and lung cancer in men and breast and lung cancer in women [5]. These findings are particularly important given that more than one in four deaths in Poland is attributable to cancer, substantially exceeding the global estimate of nearly one in six deaths. This corresponds to approximately 100,000 cancer-related deaths annually in a country with a population of over 37 million [6].

Gastrointestinal cancers are frequently associated with malnutrition, which has a profound impact on treatment tolerance, worsens clinical outcomes and prognosis, increases the risk of complications, prolongs hospital stays, and contributes to higher healthcare costs [7,8]. Antasouras et al. [9] reported that malnutrition is a common and serious problem among patients with esophageal and pharyngeal cancers. These malignancies often substantially impair food intake, frequently resulting in severe malnutrition. The relationship between nutritional status and cancer is bidirectional: nutritional status influences the course of the disease and treatment outcomes, while the disease itself adversely affects nutritional status, creating an additional challenge in cancer management. Consequently, increasing attention has been directed toward the role of appropriate nutritional management and dietary support as integral components of comprehensive cancer care. Nutritional knowledge, defined as knowledge of healthy eating principles and the ability to adapt dietary practices to an individual’s current health status, may significantly influence disease progression as well as the effectiveness of anticancer therapy [7,10].

Treatment strategies for gastrointestinal cancer may include surgery, radiotherapy, systemic chemotherapy, targeted therapy, or immunotherapy, selected according to the specific tumor type and clinical context [11]. These treatments may cause symptoms such as nausea, vomiting, mucositis, diarrhea, dysgeusia, anorexia, and fatigue, which can reduce oral intake and contribute to weight loss [7]. Patients undergoing gastrointestinal surgery may also experience impaired digestion, malabsorption, altered bowel function, or other procedure-specific nutritional consequences [12]. In patients with advanced malignancies and poor prognosis, clinicians may implement palliative care, with the primary aim of improving patients’ quality of life and alleviating disease-related symptoms [13,14]. The specific nutritional and functional consequences of these treatments vary according to tumor location and disease stage, and may in turn adversely affect patients’ overall quality of life.

Quality of life remains a fundamental component of comprehensive oncology care. The debilitating nature of cancer and the intensive treatment process substantially impair patients’ physical and psychological well-being, negatively affecting their daily functioning and overall quality of life [15]. Therefore, assessing nutritional knowledge, dietary habits, and quality of life among patients with gastrointestinal cancers appears particularly important, as does evaluating opportunities to improve nutrition education and dietary care in order to enhance the effectiveness of oncological treatment and promote patients’ well-being [7,16,17]. Nutrition-related quality-of-life instruments have recently been developed, including the CANUT-QVA, a questionnaire specifically designed to evaluate nutrition-related quality of life in patients with cancer [18]. The present study selected the WHOQOL-BREF because it is a generic, multidimensional instrument that permits assessment and comparison across physical, psychological, social, and environmental domains, is available in a validated Polish version, and has an established scoring procedure enabling comparison with a wide body of prior oncology research, rather than focusing specifically on nutrition-related quality of life. Despite growing recognition of the interplay between nutrition and quality of life in oncology, few studies have jointly examined nutritional knowledge, dietary habits, and quality of life within a gastrointestinal cancer population specifically—a group for which nutritional consequences are particularly pronounced given the anatomical and functional role of the gastrointestinal tract in food intake and digestion. The WHOQOL-BREF and QEB questionnaires were selected because they enable multidimensional assessment of the constructs central to this study’s research questions—quality of life across physical, psychological, social, and environmental domains, and nutritional knowledge, respectively—and because the Polish-language QEB was specifically developed for use in Polish nutritional research [19].

This exploratory cross-sectional survey describes dietary habits, nutritional knowledge, dietary counseling use, and multidimensional quality of life among a convenience sample of patients with gastrointestinal cancers. The primary contribution of this study lies in its description of dietary behavior and nutritional counseling use among a Polish sample of patients with heterogeneous gastrointestinal malignancies, together with an exploratory characterization of WHOQOL-BREF domains in this population, rather than in establishing the effect of gastrointestinal cancer on quality of life, which is already well established in the literature. The aim of the present study was to assess nutritional knowledge, dietary habits, and quality of life among patients with gastrointestinal cancers using data collected with the QEB and WHOQOL-BREF questionnaires, supplemented with additional author-developed questions. As this study surveyed patients with a heterogeneous range of gastrointestinal malignancies, it was designed as a small, descriptive, exploratory survey rather than a study intended to detect differences between specific cancer types. The primary objective was purely descriptive: to characterize nutritional knowledge, dietary habits, and quality of life in this population without formal hypothesis testing. Secondary objectives involved hypothesis-testing analyses—examining differences in these measures by sex, educational attainment, and age, as well as testing predefined proportions for dietary counseling use and post-diagnosis dietary change—but given the small sample size and the absence of correction for multiple comparisons, these should be interpreted as exploratory and hypothesis-generating rather than confirmatory.

We hypothesized that patients with gastrointestinal cancers would report lower quality of life, particularly in the physical domain; that dietary habits would change following diagnosis; and that nutritional knowledge and dietary behavior would differ according to sociodemographic characteristics, including sex and educational attainment.

## 2. Materials and Methods

### 2.1. Study Design

This cross-sectional, observational study included 52 participants, comprising 35 women and 17 men. Eligible participants were adults (≥18 years of age) with a self-reported diagnosis of gastrointestinal cancer. In this study, the term “gastrointestinal cancer” was used to include malignancies of the oral cavity, tongue, esophagus, stomach, small intestine, colon, rectum, liver, gallbladder, and pancreas, as reported by the participants. The exclusion criteria were age below 18 years and the absence of a diagnosis of gastrointestinal cancer. Cancer type was based on patient self-report using terminology provided in the questionnaire; “colorectal cancer” was offered as a distinct response option alongside “colon cancer” and “rectal cancer”, reflecting how patients described their own diagnosis rather than a formal clinical subsite classification. Data were collected using a questionnaire developed based on the QEB and WHOQOL-BREF instruments and supplemented with additional author-developed questions regarding changes in dietary habits and the use of dietary counseling following a cancer diagnosis.

The questionnaire was administered electronically using Google Forms (Mountain View, CA, USA) and distributed through online support groups for patients with cancer on the Meta Platforms, Inc. (Menlo Park, CA, USA) Facebook platform. The questionnaire consisted of three sections. The first section collected participants’ sociodemographic characteristics. The second section assessed nutritional knowledge and was based on the QEB questionnaire. The third section evaluated participants’ quality of life using the WHOQOL-BREF questionnaire. The survey included both single-choice and multiple-choice questions, while responses in part of the third section were recorded using the rating scale specified in the WHOQOL-BREF instrument. Data were collected between 1 March and 31 March 2024. As the survey was designed to be fully anonymous, no mechanism (e.g., login requirement, email verification, or IP tracking) was used to prevent duplicate submissions from the same individual, as this would have compromised anonymity. The exact number of Facebook groups through which the questionnaire was distributed and the approximate number of individuals exposed to the recruitment invitation were not tracked. Participation in the study was voluntary, and all responses were collected anonymously. As the questionnaire required a response to each item before submission, no missing item-level data occurred within completed responses.

### 2.2. Research Instruments

The study employed the QEB and WHOQOL-BREF questionnaires, supplemented with additional author-developed questions concerning changes in dietary habits and the use of dietary counseling following a cancer diagnosis.

#### 2.2.1. Assessment of Dietary Habits and Nutritional Knowledge

Selected dietary habits, food consumption frequency, and nutritional knowledge were assessed using the QEB questionnaire, which served as the precursor to the KomPAN questionnaire for the assessment of dietary beliefs and eating habits. The QEB questionnaire includes items addressing dietary habits, food consumption frequency, opinions on food and nutrition, self-assessment of dietary practices, nutritional knowledge and its sources, as well as respondents’ personal characteristics [19]. The QEB items assessed participants’ current dietary habits and nutritional knowledge at the time of the survey, whereas changes in dietary habits since diagnosis were assessed separately using the author-developed questions described above.

Nutritional knowledge was assessed using a 25-item scale included in the QEB questionnaire. Each statement was answered using one of three response options: “I agree,” “I disagree,” or “I have no opinion.” Because the statements vary considerably in difficulty, analysis of all 25 items is recommended. The inclusion of all statements enables reliable discrimination between respondents with insufficient, satisfactory, and good levels of nutritional knowledge [19]. Each correct response was awarded one point, whereas incorrect responses and the answer “I have no opinion” received zero points. The total nutritional knowledge score was calculated by summing the points across all items. Possible scores ranged from 0 to 25, with higher scores indicating greater nutritional knowledge. Nutritional knowledge was categorized according to the following criteria:

- Insufficient knowledge: 0–8 points;
- Satisfactory knowledge: 9–16 points;
- Good knowledge: 17–25 points.

In addition to the 25-item objective knowledge score described above, respondents were asked to rate their own nutritional knowledge using a single self-assessment item on a four-point ordinal scale (1 = insufficient, 2 = satisfactory, 3 = good, 4 = very good). The analyses examining the association between educational attainment and nutritional knowledge are based on this self-assessed rating rather than the summed objective score. Item-level responses to the 25-item objective knowledge score were collected; however, computation of a participant-level composite score was not part of the pre-specified analysis plan, and the association between education and nutritional knowledge reported in this manuscript is based on the self-assessed rating.

#### 2.2.2. Assessment of Quality of Life

Quality of life was assessed using the WHOQOL-BREF questionnaire, the abbreviated version of the WHOQOL-100 instrument. This questionnaire is designed to evaluate the quality of life of both healthy individuals and patients and is widely used in research and clinical practice [20]. Although nutrition-specific quality-of-life instruments such as the CANUT-QVA have been developed [18], the present study used the Polish WHOQOL-BREF because the objective was to assess multidimensional general quality of life across physical, psychological, social, and environmental domains. The Polish version used in this study was the culturally adapted and validated version reported by Wołowicka and Jaracz [21]. The WHOQOL-BREF consists of 26 items covering four domains of quality of life: physical health (7 items), psychological health (6 items), social relationships (3 items), and environment (8 items). In addition, the questionnaire includes two items that are analyzed separately: one assessing the respondent’s overall perception of quality of life and the other evaluating their overall perception of health. All questions refer to the four weeks preceding the survey.

The WHOQOL-BREF assesses quality of life across four domains:

1. Physical health (Domain 1)—includes activities of daily living, dependence on medicinal substances and medical aids, energy and fatigue, pain and discomfort, sleep and rest, and work capacity.

2. Psychological health (Domain 2)—encompasses body image and appearance, positive and negative feelings, self-esteem, spirituality, religion and personal beliefs, as well as thinking, learning, memory, and concentration.

3. Social relationships (Domain 3)—includes personal relationships, social support, and sexual activity.

4. Environment (Domain 4)—comprises financial resources, physical safety and security, freedom, access to and quality of health and social care, home environment, opportunities for acquiring new information and skills, participation in and opportunities for recreation and leisure activities, the physical environment (e.g., pollution, noise, traffic, and climate), and transportation [21].

Prior to score calculation, negatively worded items were reverse-coded in accordance with the standard WHOQOL-BREF scoring procedure. For each respondent, item scores within each WHOQOL-BREF domain were summed according to the standard WHOQOL-BREF scoring procedure [22]. Mean raw domain scores are presented in Table 1. These raw domain scores were then transformed to a 4–20 scale and subsequently to a 0–100 scale, following WHO guidelines [22].

This transformation allows direct comparison of WHOQOL-BREF scores with those obtained using the WHOQOL-100 instrument. The Polish version of the questionnaire was culturally adapted and validated by Wołowicka and Jaracz [21].

WHOQOL-BREF domain scores are reported as continuous variables (mean ± SD) rather than categorized into low/moderate/high classifications, as available thresholds for such categorization were derived from a non-oncology (diabetes) population [23] and have not been validated for patients with gastrointestinal cancer.

### 2.3. Statistical Analysis

Consistent with the primary and secondary objectives outlined in the Introduction, the primary outcomes were the descriptive quality-of-life and nutritional knowledge scores, while sex-, education-, and age-based comparisons, as well as dietary counseling and dietary-change proportions, were treated as secondary, exploratory outcomes. All statistical analyses were performed using R, version 4.2.2 (R Core Team) (R Foundation for Statistical Computing, Vienna, Austria.). Descriptive statistics, including the mean and standard deviation, as well as correlation analyses, were used to summarize quantitative variables. The normality of data distribution was assessed using the Shapiro–Wilk test. As the normality assumption was not met, correlations between quality-of-life domains assessed with the WHOQOL-BREF questionnaire were examined using Spearman’s rank correlation coefficient. Correlation coefficients were interpreted as follows: 0.00–0.10, negligible correlation; 0.10–0.39, weak correlation; 0.40–0.69, moderate correlation; 0.70–0.89, strong correlation; and ≥0.90, very strong correlation [24]. Because six pairwise domain correlations were tested, a Bonferroni correction was applied to control for multiple comparisons, yielding an adjusted significance threshold of α = 0.0083 (0.05/6). Categorical variables were summarized using frequencies and percentages. Differences in proportions were assessed using one- or two-sample proportion tests based on the χ^2^ statistic without Yates’ continuity correction or, when appropriate, Fisher’s exact test, with the significance level set at α = 0.05. Differences in dietary behaviors by sex were assessed using Pearson’s χ^2^ test or Fisher’s exact test, selected based on expected cell frequencies for each comparison. Exact *p*-values are reported for the primary comparisons discussed in the Results; *p*-values for the remaining comparisons were not calculated, consistent with the exploratory framing of this analysis described above. One-sided one-sample proportion tests were performed to estimate confidence intervals for predefined proportions and to determine *p*-values. The one-sided approach was chosen because the null hypothesis for this test was formulated directionally, as specified in the Methods (the null hypothesis assumed that the true proportion was less than or equal to 50%). For transparency, we additionally report the corresponding two-sided *p*-value for the dietary-improvement comparison (two-sided *p* ≈ 0.052), which likewise does not reach conventional statistical significance. Given the exploratory nature of this study and the large number of subgroup comparisons performed relative to the sample size, no correction for multiple comparisons was applied to the analyses presented in Table 2, Table 3, Table 4 and Table 5, and effect sizes and 95% confidence intervals were not systematically calculated for these comparisons; these findings should therefore be interpreted as exploratory and hypothesis-generating rather than confirmatory. The association between respondents’ educational level and self-reported nutritional knowledge was evaluated using Pearson’s χ^2^ test without Yates’ continuity correction. The analysis included respondents’ nutritional knowledge level, expressed on a four-point ordinal scale (1 = insufficient, 2 = satisfactory, 3 = good, 4 = very good), as well as changes in dietary habits following a cancer diagnosis. A *p*-value of <0.05 was considered statistically significant.

During the preparation of this manuscript, the authors used ChatGPT (GPT-5.5 Instant, OpenAI) to assist with translation of the text from Polish to English. All AI-assisted content was reviewed and edited by the authors, who take full responsibility for the content of this publication.

## 3. Results

### 3.1. Patient Characteristics

A total of 52 patients participated in the study, with women accounting for a greater proportion than men (67.3% vs. 32.7%). The largest age group comprised participants aged 41–60 years (*n* = 21, 40.4%), followed by those aged 31–40 years (*n* = 16, 30.8%) and those aged over 60 years (*n* = 11, 21.1%); the smallest group consisted of respondents aged 21–30 years (*n* = 4, 7.7%). No respondents aged 18–20 years were represented among the study participants; the youngest reported age group was 21–30 years (*n* = 4, 7.7%). Half of the participants (50.0%) had attained a higher education degree.

**Figure 1:**
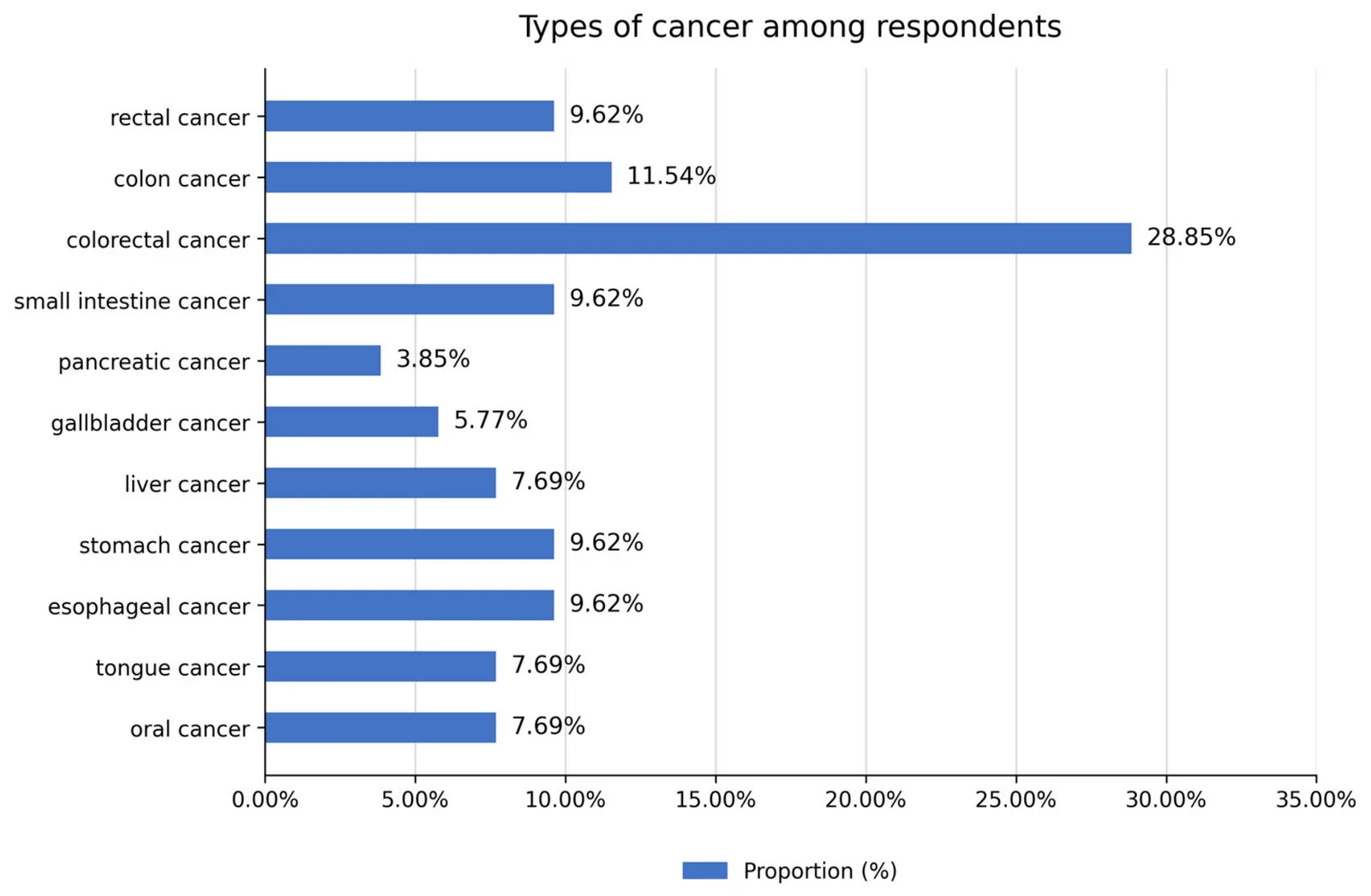
Types of cancer among respondents (*n* = 52). Percentages sum to more than 100% because six participants (11.5%) reported two concurrent gastrointestinal malignancies and were counted in each applicable category. “Colorectal cancer” reflects patients’ self-reported diagnosis without further specification of subsite; “colon cancer” and “rectal cancer” were reported separately by patients who specified the subsite. Combined, cancers of the colon and rectum (including unspecified colorectal cases) accounted for 44.8% of the 58 reported diagnoses (26 of 58).

Six participants (11.54%) were diagnosed with two gastrointestinal malignancies. Colorectal cancer (subsite not further specified by the patient) was the most frequently reported diagnosis, affecting 28.85% of the study population (*n* = 15). Colon cancer was the second most frequently reported malignancy (11.54%; *n* = 6). Esophageal, gastric, small intestinal, and rectal cancers were each reported by 9.62% of participants (*n* = 5). Oral cavity, tongue, and liver cancers were each reported by four participants (7.69%). Gallbladder cancer was reported by 5.77% of respondents (*n* = 3), whereas pancreatic cancer was the least common diagnosis, affecting 3.85% of participants (*n* = 2) (Figure 1).

### 3.2. Assessment of Patients’ Quality of Life

Regarding the overall assessment of quality of life, the largest proportion of patients rated their quality of life as “good” (38%; *n* = 20), followed by “neither good nor poor” (33%; *n* = 17), “poor” (15%; *n* = 8), very good (14%; *n* = 7) and “very poor” (0%; *n* = 0), according to the categories defined in the methodology.

When asked about their overall satisfaction with life, most participants reported being “neither satisfied nor dissatisfied” (38.5%). The remaining respondents described themselves as “satisfied” (19.2%), “very satisfied” (19.2%), “dissatisfied” (17.3%), and “very dissatisfied” (5.8%).

The results for each WHOQOL-BREF domain are presented as means and standard deviations for both the raw domain scores and the linearly transformed scores on the 0–100 scale. These results are presented in Table 1.

**Figure 2:**
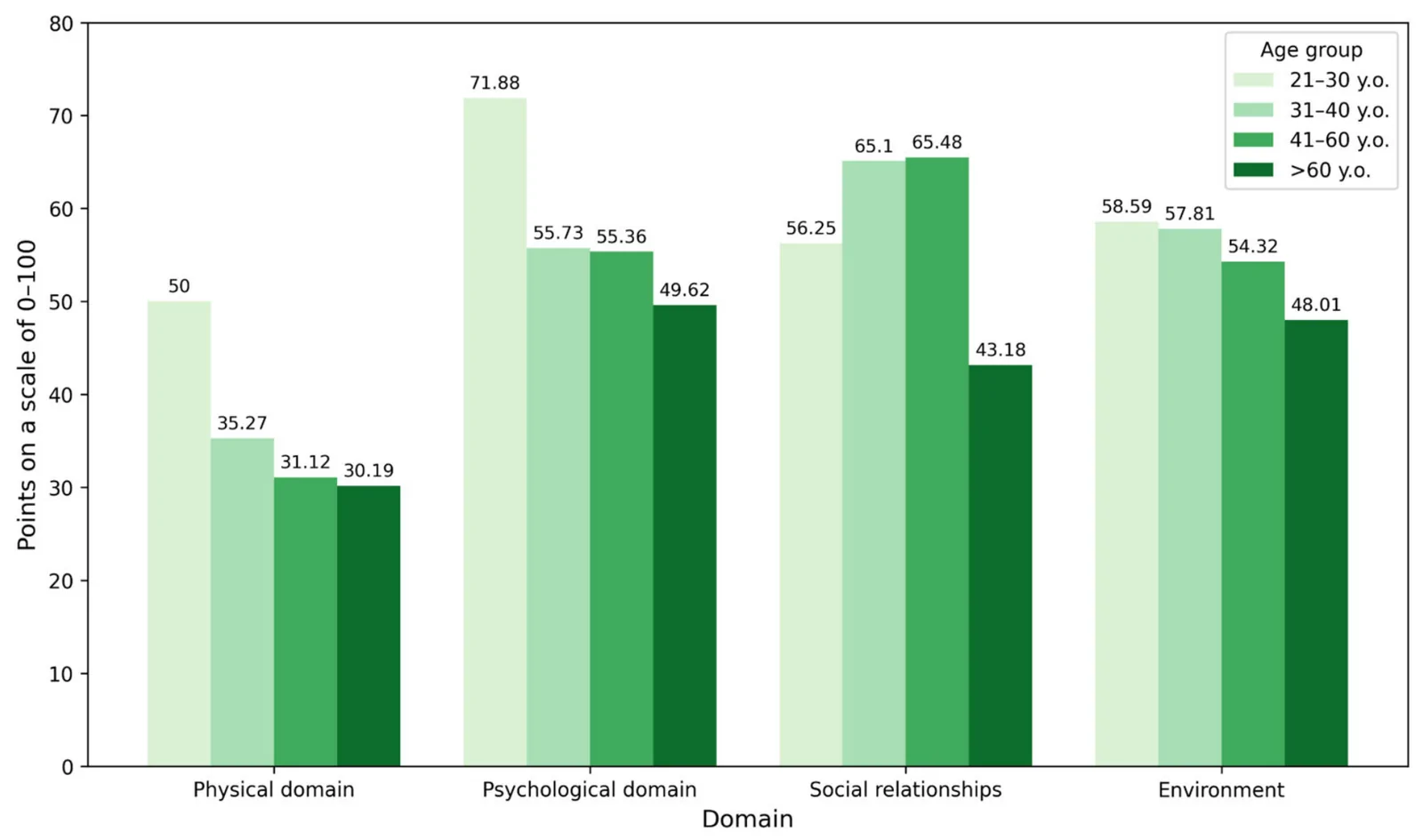
Quality of life according to age group and WHOQOL-BREF domain.

Analysis of quality-of-life scores according to age group showed that the highest score in the psychological health domain was observed among the youngest participants (21–30 years), with a mean score of 71.88 points. Participants aged over 60 years exhibited the lowest scores across all assessed domains. Within this age group, the highest mean score was recorded for the psychological health domain (49.62 points), whereas the lowest was observed for the physical health domain (30.19 points). Participants aged 31–40 and 41–60 years demonstrated relatively similar scores across the individual domains, although minor differences were observed between the two groups. A detailed summary of the results is presented in Figure 2.

The mean physical-domain WHOQOL-BREF score was 33.65 (SD = 18.31) among both women and men. Women scored, on average, 16.59 points higher than men in the psychological health domain (0–100 scale); this difference was not formally tested for statistical significance and should be interpreted descriptively. No strong correlations (i.e., ρ ≥ 0.70) were identified between the analyzed quality-of-life domains, based on the classification of Schober et al. [24]. All correlations were statistically significant (*p* < 0.05 for all pairs except the physical–social relationships domains, *p* = 0.044), with the strongest association observed between the psychological health and physical health domains (ρ = 0.69, moderate correlation). After applying a Bonferroni correction for multiple comparisons (adjusted α = 0.0083), all correlations remained statistically significant except for the association between the physical health and social relationships domains (*p* = 0.044), which did not survive correction. These findings reflect an interrelationship between the physical and psychological health domains rather than evidence that one domain influences the other. These findings are presented in Table 6.

The mean physical-domain WHOQOL-BREF score was 33.65 (SD = 18.31) on the 0–100 scale, compared with 55.53 (SD = 23.23) for psychological health, 59.94 (SD = 22.99) for social relationships, 54.39 (SD = 15.36) for environment, 62.5 (SD = 22.96) for overall quality of life, and 57.21 (SD = 28.58) for overall health. Among the surveyed patients, the physical health domain had the lowest mean score relative to the other domains assessed.

### 3.3. Patients’ Nutritional Knowledge

The study sought to determine whether patients with gastrointestinal cancers used dietary counseling services and whether they had improved their dietary habits following their cancer diagnosis. The null hypothesis was based on an expected success proportion of 50%, which was specified as the reference value for the analysis. Specifically, the null hypothesis assumed that the proportion of successful outcomes was less than or equal to 50%. The results of the analysis are presented in Table 7.

We note that the choice of 50% as the null proportion for these tests, while methodologically standard for one-sample proportion tests, is somewhat arbitrary and has limited clinical meaning; these findings are therefore best interpreted as descriptive prevalence estimates with accompanying confidence intervals, rather than as tests against a clinically meaningful threshold. Because a one-sided one-sample proportion test was used, results were considered statistically significant at *p* < 0.025 (equivalent to a two-sided significance level of α = 0.05). A majority of participants reported modifying their dietary habits after diagnosis (63.5%; *n* = 33), although this proportion was not statistically significantly greater than 50% (one-sided *p* = 0.0261 > 0.025; two-sided *p* ≈ 0.052). Only one participant reported that their dietary habits had worsened. With regard to the hypothesis concerning the use of dietary counseling services, 77% of respondents reported that they had not consulted a dietitian. The *p*-value was <0.0001, leading to rejection of the null hypothesis in favor of the alternative hypothesis that the proportion of participants who did not use dietary counseling services exceeded 50%.

The relationship between educational attainment and nutritional knowledge was also examined. Respondents’ nutritional knowledge was assessed using a four-point scale (1 = insufficient, 2 = satisfactory, 3 = good, and 4 = very good), together with self-reported changes in dietary habits following a cancer diagnosis. Associations were analyzed using Pearson’s χ^2^ test without Yates’ continuity correction, with statistical significance set at *p* < 0.05. The results are presented in Table 2.

Because the numbers of participants with primary and lower secondary education were too small to be considered representative, reliable statistical comparisons for these two groups were not possible. Most of the observed differences were not statistically significant. The only exception was the group of participants with secondary education, for whom Fisher’s exact test yielded a *p*-value of 0.051 when compared with participants holding higher education, which does not reach conventional statistical significance (Table 3).

However, the group of participants with secondary education was approximately half the size of the higher education group. Moreover, 54% of participants with secondary education reported no changes in their dietary habits following diagnosis, compared with only 27% of those with higher education, whereas only 38% reported improving their dietary habits, compared with 73% of participants with higher education. Therefore, an additional analysis was performed by combining all educational levels below higher education into a single comparison group. More than half of the participants with higher education (61.5%) rated their nutritional knowledge as at least good (score ≥ 3). In contrast, the majority of participants with primary, lower secondary, vocational, or secondary education (approximately 69%) rated their nutritional knowledge as insufficient or satisfactory (scores 1–2). This difference between participants with higher education and those with lower educational attainment corresponded to a risk difference of 30.8 percentage points (95% CI: 5.0–56.5); given the small sample size, this estimate carries substantial uncertainty and should be interpreted as exploratory rather than confirmatory (Table 4). No statistically significant differences were observed between these groups with respect to changes in dietary habits following a cancer diagnosis.

To evaluate the extent to which respondents’ dietary habits were consistent with general nutritional recommendations for patients with cancer and to compare dietary behaviors between women and men, selected aspects of the participants’ diets were analyzed. These included meal regularity, snacking between meals, consumption of fast food and fried foods, choice of dairy products, frequency of fish, legume, fruit, and vegetable consumption, as well as alcohol intake. Statistical significance was assessed using Pearson’s χ^2^ test without Yates’ continuity correction or Fisher’s exact test, as appropriate. Several differences in dietary patterns between women and men were observed, although most did not reach statistical significance. Women were more likely to consume meals at regular times (51% vs. 18%; *p* = 0.058), reported eating fish several times per week more frequently (43% vs. 35%; *p* = 0.14), consumed fruit several times per day more often (54% vs. 18%; *p* = 0.034), and reported eating vegetables several times per day more frequently (91% vs. 76%; *p* = 0.11). In addition, a higher proportion of women consumed at least four servings of fruit and vegetables per day (69% vs. 29%), whereas men most commonly reported consuming three servings per day (47%; *p* = 0.022). Women also reported complete abstinence from alcohol more frequently than men (69% vs. 29%), whereas men were more likely to consume alcohol one to three times per month (41% vs. 29%). The largest sex-based differences were observed in fruit consumption frequency (risk difference: 36 percentage points; 95% CI: 11.4–60.6) and daily fruit-and-vegetable servings (risk difference: 40 percentage points; 95% CI: 13.5–66.5); given the small sample size and the number of comparisons performed, these estimates carry substantial uncertainty and should be interpreted as exploratory rather than confirmatory (Table 5).

Analysis of the dietary habits of patients with gastrointestinal cancers showed that 40% of the overall study population consumed all meals at regular times, while an additional 37% reported eating at least some meals at regular times. However, the majority of participants (67%) reported snacking between meals. Furthermore, 52% consumed fried foods several times per week, and 52% reported consuming whole-fat milk and dairy products, whereas 46% preferred reduced-fat dairy products. Participants also reported frequent consumption of fruit and vegetables. Approximately 66% consumed either three or four servings of fruit and vegetables per day. Moreover, 87% reported eating vegetables several times per day, while 88% consumed fruit at least once daily (46% once daily and an additional 42% several times daily). Most respondents also indicated that they did not add sugar to hot beverages. In addition, the majority reported abstaining from energy drinks (69%) and alcohol (56%), and 42% stated that they did not consume fast food. Overall, these findings describe a mixed pattern of self-reported dietary behaviors: frequent fruit and vegetable consumption alongside a high prevalence of snacking between meals (67%), frequent consumption of fried foods (52% several times per week), predominant use of full-fat dairy products (52%), and relatively infrequent legume consumption (only 23% several times per week). As this study assessed only self-reported consumption frequency rather than quantitative food, energy, protein, or micronutrient intake, these findings should be interpreted as descriptive of self-reported dietary behaviors rather than as an objective assessment of nutritional adequacy or formal adherence to nutritional recommendations (Table 5).

**Figure 3:**
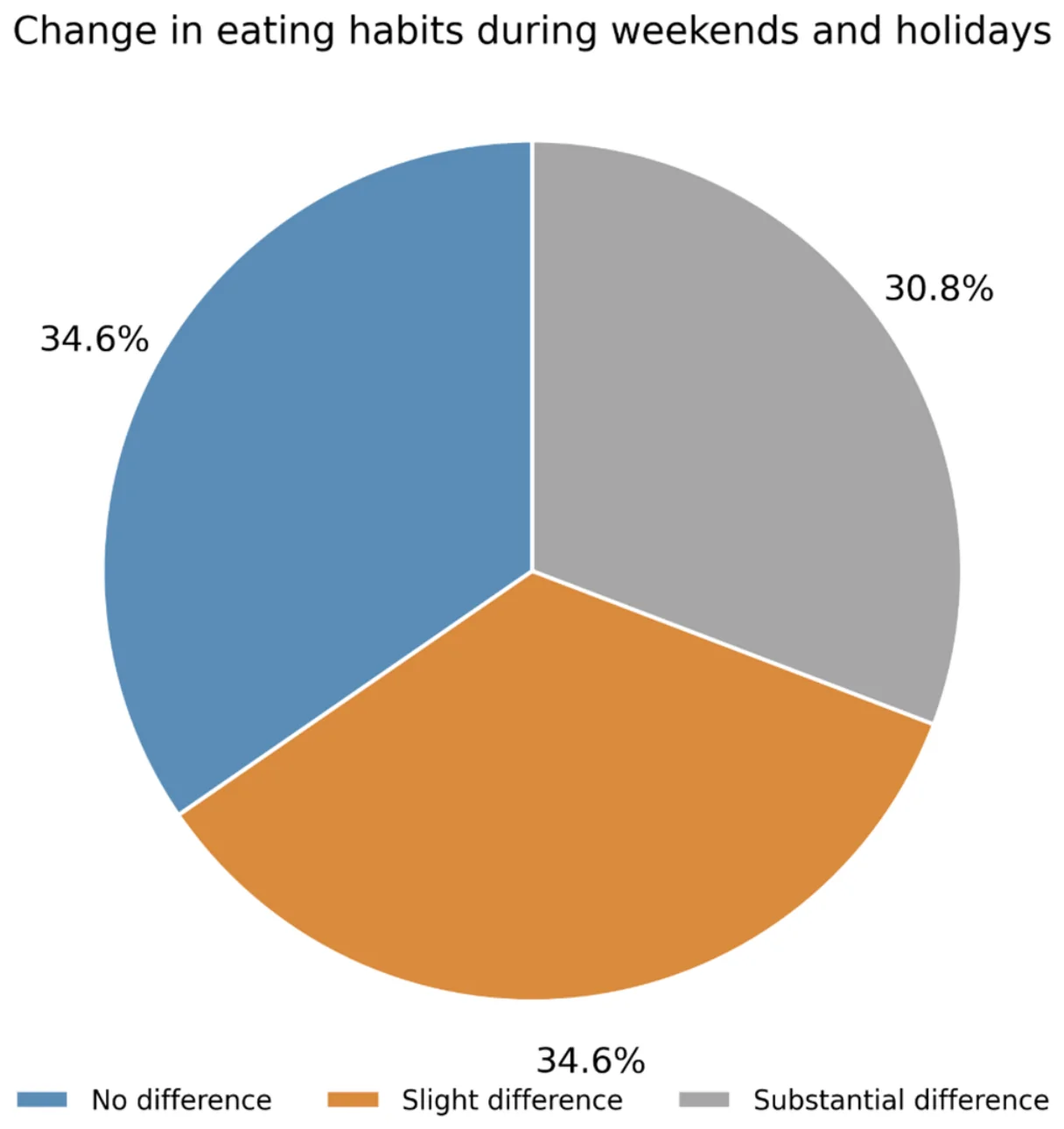
Changes in respondents’ dietary habits on weekends and public holidays compared with weekdays.

Responses regarding changes in dietary habits on weekends and public holidays compared with weekdays were relatively evenly distributed. Overall, 34.6% of respondents reported that their dietary habits did not differ, an equal proportion (34.6%) indicated that their dietary habits differed only slightly, whereas a slightly smaller proportion (30.8%) reported substantial differences in their eating patterns on weekends and public holidays (Figure 3).

**Figure 4:**
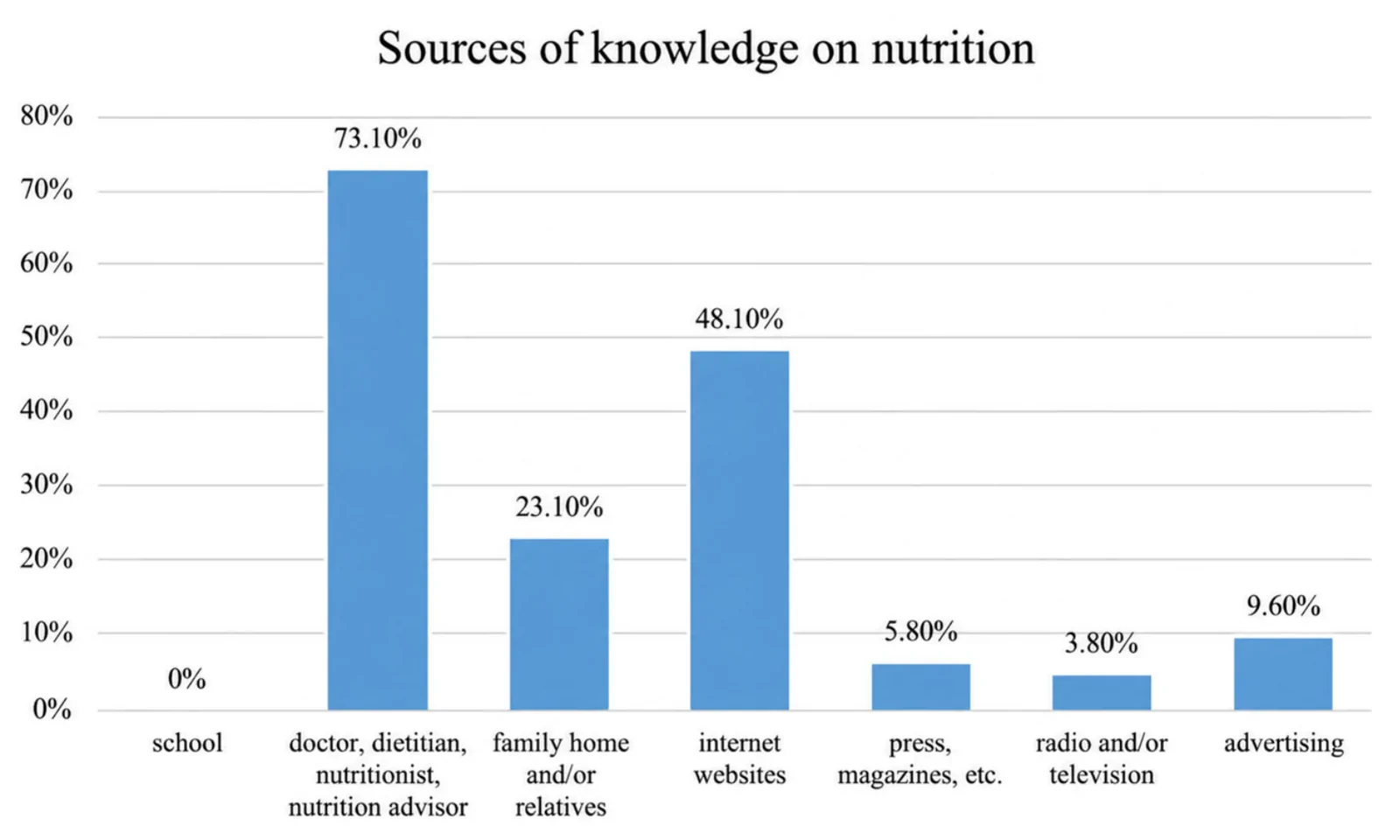
Sources of respondents’ nutritional knowledge (*n* = 52). Respondents could select multiple sources; percentages therefore sum to more than 100% and should not be interpreted as mutually exclusive.

The majority of respondents reported obtaining nutritional information from physicians, dietitians, or nutrition counselors (73.1%, *n* = 38). The Internet (48.1%, *n* = 25) and family members or relatives (23.1%, *n* = 12) were the next most frequently reported sources of nutritional information (Figure 4).

Full sociodemographic characteristics of the study population are presented in Table 8.

## 4. Discussion

Several of the comparative studies discussed below were conducted in breast, lung, cervical, or mixed cancer populations rather than in patients with gastrointestinal cancer specifically. Because tumor site, treatment exposure, gastrointestinal symptoms, and nutritional mechanisms differ substantially across cancer populations, these comparisons should be considered indirect and interpreted with caution, rather than as direct evidence that the same associations apply to gastrointestinal cancer.

This study found that, among the surveyed patients with gastrointestinal cancers, the physical health domain had the lowest WHOQOL-BREF score, with moderate scores across the psychological, social, and environmental domains; that most participants had not received dietary counseling despite reporting improvements in their dietary habits following diagnosis; and that nutritional knowledge and dietary behavior differed by sex and educational attainment. Quality of life in patients with cancer is widely recognized as an important factor in treatment and follow-up care [25], and a 2025 analysis of 16,210 patients across 17 cancer types demonstrated that quality-of-life measures are predictive of survival [26].

These findings highlight the importance of quality of life as a key outcome in both inpatient and outpatient oncology care. The analysis of overall quality of life and life satisfaction showed that nearly half of the participants (48%; 0% very poor, 15% poor, and 33% neither poor nor good) rated their quality of life between 1 and 3. This finding is consistent with evidence from patients with gastrointestinal cancer specifically. In colorectal cancer survivors, nutritional status has been shown to be significantly associated with quality of life [27,28]. Similarly, studies in patients with gastric cancer [29] and pancreatic cancer [30] have reported that poorer nutritional status is associated with lower quality of life, underscoring the relevance of nutrition-focused care across gastrointestinal cancer subtypes. These findings are consistent with previous studies reporting a high prevalence of impaired psychological well-being among patients with cancer. In the study by Lewandowska et al., 47% of respondents reported elevated levels of anxiety, while 30% acknowledged experiencing depressive symptoms, both of which may substantially reduce quality of life [31]. Similarly, another study found that approximately 40% of patients with cancer experienced mood disorders that adversely affected their daily functioning and overall well-being [32]. Mziray et al. reported that 45.8% of participants experienced moderate anxiety, whereas depressive symptoms were observed in all study participants, with 45.8% meeting the criteria for moderate depression [33]. Pasek et al. also demonstrated relatively low quality of life among women with cervical cancer before radiotherapy, followed by a marked improvement after completion of treatment [34]. The present findings are further supported by a 2023 systematic review, which concluded that the quality of life of patients with cancer is substantially impaired, particularly with respect to physical, social, and emotional functioning. The authors emphasized that quality of life is influenced not only by the symptoms of the disease itself but also by the burden associated with anticancer treatment, underscoring the need for comprehensive supportive care and effective symptom management to improve patients’ overall well-being [35].

These findings are consistent with the above-mentioned systematic review, in which the physical domain was also identified as the most severely impaired aspect of quality of life among patients with cancer [35]. In the study by Lewandowska et al., 59% of respondents reported having to reduce their daily physical activity, while 41% were unable to work because of their illness. Furthermore, 49% of participants required temporary assistance with daily functioning [31]. By comparison, women with breast cancer included in another study achieved a mean physical health domain score of 62.5 points, almost twice as high as that observed in the present study [36].

Quality-of-life scores in the social relationships, psychological health, and environmental domains were relatively similar, with none exceeding 60 points. In the study by Lewandowska et al., 37% of respondents reported that their relationships with family and friends had not changed as a result of their illness, whereas 15% indicated that cancer had actually strengthened these relationships [31]. Only 20% reported becoming more socially withdrawn from their loved ones. Another study reported slightly higher scores across these domains, with mean values of 66.6 points for the psychological domain, 63.4 points for the environmental domain, and the highest score of 73.2 points for the social relationships domain [36]. Social functioning plays a crucial role in the well-being of patients with cancer, as confirmed by a study published in 2026. Higher levels of perceived and received emotional and instrumental support were associated with lower levels of anxiety and depressive symptoms, as well as better quality of life. These findings indicate that well-developed social support systems may represent an important component of improving psychological well-being and promoting more effective coping strategies among patients with cancer [37]. Furthermore, Bossi et al. [38] emphasized the need to increase awareness among institutional stakeholders and healthcare funding bodies regarding the importance of nutritional care in oncology, as well as to allocate greater financial and human resources to the management of nutrition-related issues in patients with cancer.

The following paragraph discusses hypothesized mechanisms drawn from the previous literature that may plausibly explain associations between nutritional knowledge, dietary behavior, and quality of life; these mechanisms were not directly tested in the present study and should be clearly distinguished from the study’s own observed findings, which are limited to the descriptive and exploratory associations reported in the Results. Several mechanisms may plausibly link nutritional knowledge, dietary behavior, and quality of life in patients with gastrointestinal cancer. First, gastrointestinal malignancies and their treatment frequently impair nutrient absorption and intake, contributing to malnutrition and sarcopenia, which are directly associated with reduced physical functioning and, consequently, lower physical-domain quality of life [27,39]. Second, greater nutrition knowledge may enhance patients’ self-efficacy. Evidence suggests that higher food and nutrition literacy is associated with greater diet-related self-efficacy among individuals with cancer [40]. Furthermore, higher self-efficacy has been linked to better psychological adjustment, more effective coping, and lower levels of distress and anxiety in oncology populations [41]. Third, greater nutritional knowledge may facilitate adherence to evidence-based dietary recommendations and nutritional interventions during cancer treatment. Better maintenance of nutritional status and skeletal muscle mass has been associated with improved physical functioning and higher health-related quality of life in patients with cancer [12,42]. These proposed mechanisms remain speculative in the context of the present cross-sectional design and would require prospective or mechanistic studies to confirm.

In the present study, no formal association was observed between participants’ sex and overall quality of life. A descriptive difference was noted in the psychological health domain, where women scored 16.59 points higher than men on average (see Results); however, as this difference was not formally tested, it should not be interpreted as evidence of a sex-based association in this domain. Lewandowska et al. [31] reported that women were significantly more likely than men to seek professional psychological or psychiatric support and were also more likely to express determination to cope with the disease and its symptoms. Similarly, a study published in 2023 demonstrated that women with cancer reported higher overall quality of life than men [25].

Regardless of cancer type, a diagnosis of malignancy appears to be an important factor motivating patients to modify their dietary behaviors, as reported in the literature [43]. In the present study, a trend toward improved dietary habits was observed among patients diagnosed with gastrointestinal cancer, and these findings are consistent with previous reports. For example, a study conducted in 2021 found that more than half of patients with cancer (55%) reported changing their dietary habits following diagnosis. The most common modifications included increased consumption of fruit and vegetables (36%) and reduced intake of red meat (25%), sugar and sweets (20%), and dietary fat (12%). A small proportion of participants (7%) also reported eliminating dairy products from their diet [44]. Clotas et al. [45] found that 9.5% of women with breast cancer adhered to healthy dietary recommendations after diagnosis, compared with 5.8% who had followed healthy dietary practices before diagnosis. Likewise, Krusińska et al. [46] demonstrated that individuals characterized by a prudent dietary pattern—marked by frequent consumption of cottage cheese, milk and fermented dairy products, fruit, vegetables, whole-grain bread, fish, and cheese—were less likely to be diagnosed with breast or lung cancer. In contrast, participants with diets characterized by frequent consumption of highly processed foods, fast food, canned products, instant soups, and alcohol were at greater risk of developing cancer. Similarly, Rim et al. [47] reported that breast cancer survivors exhibited healthier dietary habits than women in the control group. A systematic review by Aldossari et al. [48] confirmed that patients often introduce beneficial dietary changes following a cancer diagnosis; however, these modifications are generally modest and inconsistent. These findings suggest that a cancer diagnosis alone is insufficient to achieve substantial and sustained improvements in dietary habits. Because the WHOQOL-BREF is a generic instrument, it may not capture nutrition-specific concerns as comprehensively as a dedicated nutrition-related quality-of-life instrument; future studies could consider using the WHOQOL-BREF together with the CANUT-QVA to more fully characterize the relationship between nutrition and quality of life in this population.

## 5. Limitations

This study has several limitations that should be considered when interpreting the findings. Cancer diagnoses, including the specific gastrointestinal subsite, were based entirely on patient self-report and were not verified against medical records; consequently, terminology such as “colorectal cancer” versus “colon cancer” or “rectal cancer” may reflect differences in how individual patients described their own diagnosis rather than a standardized clinical classification, and misclassification cannot be excluded.

This study grouped several biologically and clinically distinct malignancies (e.g., esophageal, gastric, colorectal, hepatobiliary, and pancreatic cancers) into a single “gastrointestinal cancer” category. These cancers differ substantially in treatment pathways, nutritional risk, and prognosis; combining them into one group may obscure clinically meaningful differences between cancer types, and the findings should be interpreted as reflecting this heterogeneous population as a whole rather than any single malignancy.

Participants were recruited exclusively through online cancer support groups on Facebook, using a convenience sampling approach. This method likely overrepresents patients who are younger, digitally literate, socially engaged, interested in nutrition and cancer-related information, and well enough to participate in an online survey, while underrepresenting patients with advanced disease, cognitive impairment, poor performance status, limited digital access, or low health literacy. The response rate, number of individuals who viewed the survey, and possible duplicate responses were not recorded. Given these substantial sources of selection bias, the findings of this study should not be generalized to the broader population of patients with gastrointestinal cancer, and should instead be interpreted as reflecting a specific, self-selected subgroup of relatively engaged and digitally accessible patients. The questionnaire was administered in Polish to participants recruited through Polish-language Facebook support groups, and age eligibility (≥18 years) was based on self-report.

This study did not collect information on cancer stage (including TNM classification), localized versus metastatic or recurrent disease status, time since diagnosis, current or previous treatment (surgery, chemotherapy, radiotherapy, or immunotherapy), treatment-related adverse effects, presence of a stoma, weight loss, nutritional-risk status, body mass index, comorbidities, performance status, or socioeconomic status. These variables are established determinants of both dietary behavior and quality of life in patients with cancer, and their absence limits our ability to interpret the observed associations or to rule out confounding. In particular, observed WHOQOL-BREF scores may be more strongly determined by unmeasured clinical factors such as disease stage, treatment burden, or comorbidities than by dietary or nutritional factors, and the present findings should not be interpreted as evidence that nutritional factors explain the observed quality-of-life scores.

With a total sample of 52 participants, several subgroup analyses were based on very small cell sizes (e.g., *n* = 2 for pancreatic cancer, *n* = 1 for lower-secondary education). No sample-size or power calculation was performed prior to data collection, resulting in a high risk of Type II error (false-negative findings), particularly for subgroup comparisons. Findings from these small subgroups should therefore be interpreted as descriptive and hypothesis-generating rather than confirmatory. Furthermore, this study involved numerous statistical comparisons across dietary habits, nutritional knowledge, and quality-of-life measures (Table 2, Table 3, Table 4 and Table 5), without correction for multiple testing. This increases the risk of false-positive findings, and statistically significant findings from these analyses should therefore be interpreted with caution. All analyses examining associations between nutritional knowledge, dietary behavior, and quality of life were univariate and unadjusted for potential confounders such as age, sex, education, cancer type, treatment status, disease stage, or time since diagnosis; given the small sample size, a multivariable model adjusting for these factors would not have been appropriate. These findings should therefore not be interpreted as evidence of associations independent of these unmeasured factors. We did not examine potential predictors of dietary counseling use (e.g., age, sex, education, cancer type, or time since diagnosis), as the small sample size would not support a reliable multivariable model; this remains an important direction for future research with larger samples.

This study employed a cross-sectional design, which allows for the description of associations but does not permit causal inference. In particular, the findings cannot establish that gastrointestinal cancer caused the observed impairment in quality of life, that dietary changes led to improved quality of life, or that dietary counseling would improve patient outcomes. The associative nature of these findings should be reflected in the interpretation of the conclusions.

Nutritional knowledge in the education-related analyses (Table 2, Table 3 and Table 4) was based on a single self-assessed item rather than the objective 25-item QEB knowledge score described in the Methods. Self-assessed knowledge may not accurately reflect actual nutritional knowledge and is subject to social desirability and self-perception bias; this distinction should be considered when interpreting the association between education and nutritional knowledge reported in this study.

As noted in the Methods, WHOQOL-BREF domain scores are reported as continuous variables rather than categorized, as available classification thresholds were derived from a non-oncology population and have not been validated for gastrointestinal cancer. Dietary habits were self-reported and assessed retrospectively for changes since diagnosis, introducing the possibility of recall bias. Because time since diagnosis was not recorded, participants may have been diagnosed months or years before survey completion, and current dietary habits may differ substantially from those immediately following diagnosis; the reported ‘change after diagnosis’ should therefore be interpreted with this additional uncertainty in mind.

## 6. Conclusions

Among the patients with gastrointestinal cancers surveyed in this study, the physical health domain had the lowest WHOQOL-BREF score, with comparatively higher, though still moderate, scores observed in the psychological, social relationships, and environmental domains. This study identified associations between selected dietary and nutritional factors—including nutritional knowledge, dietary behavior, and use of dietary counseling—and reported quality of life in this sample; longitudinal and intervention studies are needed to determine the directionality and clinical significance of these associations. Women reported more favorable dietary behaviors than men in selected measures (fruit consumption and daily fruit-and-vegetable servings), whereas participants with higher educational attainment exhibited the highest self-assessed levels of nutritional knowledge. These findings should not be generalized beyond the studied sample, given its convenience-based recruitment and modest size, and should be interpreted in light of the use of a generic quality-of-life instrument and exploratory, non-cancer-derived classification thresholds.

## Figures and Tables

**Table 1:** Mean values and standard deviations of the WHOQOL-BREF domain scores.

Domain	Raw Score (±SD) ^a^	Mean (±Standard Deviation) [Scale 0–100]
Physical domain	16.42 (±5.13)	33.65 (±18.31)
Psychological domain	19.33 (±5.58)	55.53 (±23.23)
Social relationships	10.19 (±2.76)	59.94 (±22.99)
Environment	25.4 (±4.92)	54.39 (±15.36)
Overall quality of life	3.5 (±0.92)	62.5 (±22.96)
Overall health	3.29 (±1.14)	57.21 (±28.58)

^a^ For the four WHOQOL-BREF domains, raw scores represent the mean of summed item scores within each domain; for the single-item measures (overall quality of life and overall health), raw scores represent the mean item rating (1–5 scale). SD = standard deviation.

**Table 2:** Distribution of self-assessed nutritional knowledge according to respondents’ educational level, with participants holding higher education serving as the reference group.

	Primary (*n* = 2)	Lower Secondary (*n* = 1)	Secondary (*n* = 13)	Vocational (*n* = 10)	Higher (*n* = 26)
Insufficient (1)	1 (50%)	1 (100%)	4 (31%)	2 (20%)	2 (7.7%)
Satisfactory (2)	1 (50%)	0	4 (31%)	5 (50%)	8 (31%)
Good (3)	0	0	3 (23%)	3 (30%)	9 (35%)
Very good (4)	0	0	2 (15%)	0	7 (27%)
Fisher’s exact test (*p*-value)	n/a (*n* = 2)	n/a (*n* = 1)	0.3	0.2	-

Fisher’s exact test *p*-values were not reported for the primary (*n* = 2) and lower-secondary (*n* = 1) education groups, as statistical tests based on such small subgroups convey little meaningful information.

**Table 3:** Distribution of self-reported changes in dietary habits following cancer diagnosis, according to respondents’ educational level (Fisher’s exact test).

	Primary (*n* = 2)	Lower Secondary (*n* = 1)	Secondary (*n* = 13)	Vocational (*n* = 10)	Higher (*n* = 26)
No change	0	1 (100%)	7 (54%)	3 (30%)	7 (27%)
Yes, worsened	0	0	1 (7.7%)	0	0
Yes, improved	2 (100%)	0	5 (38%)	7 (70%)	19 (73%)
Fisher’s exact test (*p*-value)	n/a (*n* = 2)	n/a (*n* = 1)	0.051	>0.9	-

Fisher’s exact test *p*-values were not reported for the primary (*n* = 2) and lower-secondary (*n* = 1) education groups, as statistical tests based on such small subgroups convey little meaningful information.

**Table 4:** Distribution of responses to the question on self-assessed nutritional knowledge according to educational level, comparing participants with higher education to those with other educational levels.

	Other Educational Levels (*n* = 26)	Higher Education (*n* = 26)	Fisher’s Exact Test *p*-Value
Insufficient (1)	8 (30.8%)	2 (7.7%)	0.026
Satisfactory (2)	10 (38.5%)	8 (30.8%)	
Good (3)	6 (23%)	9 (34.6%)	
Very good (4)	2 (7.7%)	7 (26.9%)	

**Table 5:** Dietary habits of the study participants by sex.

Variable	Total (*n* = 52)	Female (*n* = 35)	Male (*n* = 17)	*p*-Value
Number of meals consumed per day				
3 meals	15 (29%)	8 (23%)	7 (41%)	
4 meals	14 (27%)	9 (26%)	5 (29%)	
5 or more meals	23 (44%)	18 (51%)	5 (29%)	
Consuming meals at regular times of the day				*p* = 0.058
No	12 (23%)	7 (20%)	5 (29%)	
Yes, some meals	19 (37%)	10 (29%)	9 (53%)	
Yes, all meals	21 (40%)	18 (51%)	3 (18%)	
Snacking between meals				*p* = 0.7
No	17 (33%)	12 (34%)	5 (29%)	
Yes	35 (67%)	23 (66%)	12 (71%)	
Frequency of fast food consumption (e.g., fries, burgers, pizza, hot dogs)				
Never	22 (42%)	16 (46%)	6 (35%)	
1–3 times per month	9 (17%)	4 (11%)	5 (29%)	
Once a week	12 (23%)	9 (26%)	3 (18%)	
Several times a week	9 (17%)	6 (17%)	3 (18%)	
Frequency of consuming fried foods (meat, flour-based)				
Never	7 (13%)	6 (17%)	1 (5.9%)	
1–3 times per month	5 (9.6%)	5 (14%)	0 (0%)	
Once a week	9 (17%)	5 (14%)	4 (24%)	
Several times a week	27 (52%)	16 (46%)	11 (65%)	
Once a day	4 (7.7%)	3 (8.6%)	1 (5.9%)	
Type of milk and dairy products most frequently consumed				
Fat-free	1 (1.9%)	1 (2.9%)	0 (0%)	
Reduced-fat	24 (46%)	17 (49%)	7 (41%)	
Full-fat (standard)	27 (52%)	17 (49%)	10 (59%)	
Frequency of consuming fermented dairy drinks (yoghurt, kefir)				
1–3 times per month	1 (1.9%)	1 (2.9%)	0 (0%)	
Once a week	4 (7.7%)	1 (2.9%)	3 (18%)	
Several times a week	22 (42%)	14 (40%)	8 (47%)	
Once a day	14 (27%)	11 (31%)	3 (18%)	
Several times a day	11 (21%)	8 (23%)	3 (18%)	
Frequency of consuming fish and fish-based dishes				*p* = 0.14
1–3 times per month	8 (15%)	4 (11%)	4 (24%)	
Once a week	15 (29%)	8 (23%)	7 (41%)	
Several times a week	21 (40%)	15 (43%)	6 (35%)	
Once a day	7 (13%)	7 (20%)	0 (0%)	
Several times a day	1 (1.9%)	1 (2.9%)	0 (0%)	
Frequency of consuming legumes (beans, peas, etc.)				
Never	1 (1.9%)	1 (2.9%)	0 (0%)	
1–3 times per month	20 (38%)	10 (29%)	10 (59%)	
Once a week	14 (27%)	11 (31%)	3 (18%)	
Several times a week	12 (23%)	8 (23%)	4 (24%)	
Once a day	4 (7.7%)	4 (11%)	0 (0%)	
Several times a day	1 (1.9%)	1 (2.9%)	0 (0%)	
Frequency of fruit consumption				*p* = 0.034
Several times a week	6 (12%)	3 (8.6%)	3 (18%)	
Once a day	24 (46%)	13 (37%)	11 (65%)	
Several times a day	22 (42%)	19 (54%)	3 (18%)	
Frequency of vegetable consumption				*p* = 0.11
Once a week	1 (1.9%)	1 (2.9%)	0 (0%)	
Once a day	6 (12%)	2 (5.7%)	4 (24%)	
Several times a day	45 (87%)	32 (91%)	13 (76%)	
Number of fruit and vegetable servings per day				*p* = 0.022
1 serving	1 (1.9%)	1 (2.9%)	0 (0%)	
2 servings	5 (9.6%)	1 (2.9%)	4 (24%)	
3 servings	17 (33%)	9 (26%)	8 (47%)	
4 servings	17 (33%)	13 (37%)	4 (24%)	
5 or more servings	12 (23%)	11 (31%)	1 (5.9%)	
Frequency of consuming sweets and confectionery				
1–3 times per month	7 (13%)	5 (14%)	2 (12%)	
Once a week	17 (33%)	12 (34%)	5 (29%)	
Several times a week	11 (21%)	7 (20%)	4 (24%)	
Once a day	7 (13%)	4 (11%)	3 (18%)	
Adding sugar to hot beverages (tea, cocoa, coffee)				
No	27 (52%)	21 (60%)	6 (35%)	
Yes, one teaspoon	18 (35%)	9 (26%)	9 (53%)	
Yes, two or more teaspoons	7 (13%)	5 (14%)	2 (12%)	
Frequency of energy drink consumption				
Never	36 (69%)	25 (71%)	11 (65%)	
1–3 times per month	9 (17%)	4 (11%)	5 (29%)	
Once a week	3 (5.8%)	2 (5.7%)	1 (5.9%)	
Several times a week	4 (7.7%)	4 (11%)	0 (0%)	
Frequency of alcohol consumption				
Never	29 (56%)	24 (69%)	5 (29%)	
1–3 times per month	17 (33%)	10 (29%)	7 (41%)	
Once a week	4 (7.7%)	1 (2.9%)	3 (18%)	
Several times a week	2 (3.8%)	0 (0%)	2 (12%)	

**Table 6:** Spearman correlation coefficients (ρ), 95% confidence intervals, and *p*-values between WHOQOL-BREF quality-of-life domains (*n* = 52).

Domain Pair	*p*	95% Cl	*p*-Value	Adjusted *p* (Bonferroni) ^b^	Strength ^a^
Physical health–Psychological health	0.69	0.51–0.81	<0.001	<0.001	Moderate
Environment–Psychological health	0.58	0.37–0.74	<0.001	<0.001	Moderate
Psychological health–Social relationship	0.54	0.31–0.71	<0.001	<0.001	Moderate
Environment–Physical health	0.50	0.26–0.68	<0.001	<0.001	Moderate
Environment–Social relationships	0.49	0.25–0.67	<0.001	<0.001	Moderate
Physical health–Social relationships	0.28	0.01–0.51	0.044	0.264	Weak

^a^ Correlation strength classified according to Schober et al. [24]: 0.00–0.10 negligible, 0.10–0.39 weak, 0.40–0.69 moderate, 0.70–0.89 strong, ≥0.90 very strong. ^b^ Adjusted for six pairwise comparisons using the Bonferroni correction (α = 0.05/6 = 0.0083).

**Table 7:** Proportion of responses regarding not using dietetic services and improvement in dietary habits after receiving a cancer diagnosis, including the 95% confidence interval. (The 95% confidence interval for dietary counseling non-use reflects a one-sided bound, consistent with the one-sided test applied to this comparison; the 95% confidence interval for dietary-improvement reflects a standard two-sided interval).

Variable	Number of Patients	Proportion	95% Confidence Interval	*p*-Value
Patients did not seek dietary counseling	40	76.9%	66.13–100%	<0.0001
Patients’ dietary habits improved	33	63.46%	50–75.3%	0.0261

**Table 8:** Sociodemographic characteristics of study participants (*n* = 52).

Variable	*n*	%
Sex		
Female	35	67.3%
Male	17	32.7%
Age group		
21–30 years	4	7.7%
31–40 years	16	30.8%
41–60 years	21	40.4%
>60 years	11	21.1%
Educational attainment		
Primary	2	3.8%
Lower secondary	1	1.9%
Secondary	13	25.0%
Vocational	10	19.2%
Higher	26	50.0%

## Data Availability

The data used to support the findings of this study can be made available by the corresponding author upon request.

## References

[1] World Health Organization Cancer. https://www.who.int/news-room/fact-sheets/detail/cancer.

[2] Siegel R.L., Kratzer T.B., Wagle N.S., Sung H., Jemal A. (2026). Cancer statistics, 2026. CA Cancer J. Clin..

[3] European Commission, Joint Research Centre European Cancer Information System: Cancer Factsheets. https://ecis.jrc.ec.europa.eu/factsheets.

[4] Siegel R.L., Giaquinto A.N., Jemal A. (2024). Cancer statistics, 2024. CA Cancer J. Clin..

[5] O Onkologii Dla Każdego. https://pacjent.gov.pl/aktualnosc/o-onkologii-dla-kazdego.

[6] Krajowy Rejestr Nowotworów Nowotwory Złośliwe w Polsce—Epidemiologia. https://onkologia.org.pl/pl/epidemiologia/nowotwory-zlosliwe-w-polsce.

[7] Muscaritoli M., Arends J., Bachmann P., Baracos V., Barthelemy N., Bertz H., Bozzetti F., Hütterer E., Isenring E., Kaasa S. (2021). ESPEN practical guideline: Clinical Nutrition in cancer. Clin. Nutr..

[8] Zhang L., Ding Z., Zhao Y., Cheng Z., Hu J., Huo L. (2025). Advances and challenges in nutritional screening and assessment for cancer patients: A comprehensive systematic review and future directions. Front. Nutr..

[9] Antasouras G., Papadopoulou S.K., Tolia M., Pandi A.L., Spanoudaki M., Tsoukalas N., Tsourouflis G., Psara E., Mentzelou M., Giaginis C. (2023). May Nutritional Status Positively Affect Disease Progression and Prognosis in Patients with Esophageal and Pharyngeal Cancers? A Scoping Review of the Current Clinical Studies. Med. Sci..

[10] Arends J. (2024). Malnutrition in cancer patients: Causes, consequences and treatment options. Eur. J. Surg. Oncol..

[11] Koustas E., Trifylli E.M., Sarantis P., Papadopoulos N., Karapedi E., Aloizos G., Damaskos C., Garmpis N., Garmpi A., Papavassiliou K.A. (2022). Immunotherapy as a Therapeutic Strategy for Gastrointestinal Cancer-Current Treatment Options and Future Perspectives. Int. J. Mol. Sci..

[12] Arends J., Bachmann P., Baracos V., Barthelemy N., Bertz H., Bozzetti F., Fearon K., Hütterer E., Isenring E., Kaasa S. (2017). ESPEN guidelines on nutrition in cancer patients. Clin. Nutr..

[13] Zasowska-Nowak A. (2022). Directions of development of palliative care based on the literature. Med. Paliatywna/Palliat. Med..

[14] Petrillo L.A., Agne J.L. (2026). Re-Examining Early in Early Palliative Care: Precedent, Reality, and Future Research Priorities. JCO Oncol. Pract..

[15] Zhabagin K., Zhabagina A., Shalgumbayeva G., Toleutayeva D., Baissalbayeva A., Toleutayev T., Telmanova Z., Igissin N., Moore M. (2024). Quality of Life of Colorectal Cancer Patients: A Literary Review. Iran. J. Public Health.

[16] Alam M.M., Rahman T., Afroz Z., Chakraborty P.A., Wahab A., Zaman S., Hawlader M.D.H. (2020). Quality of Life (QoL) of cancer patients and its association with nutritional and performance status: A pilot study. Heliyon.

[17] Folwarski M.A. (2023). Quality of life as an important goal of therapy for cancer patients on home enteral nutrition. Nowotw. J. Oncol..

[18] Drareni K., Mercier C., Riantiningtyas R., Dougka A., Riche B., Roux P., Fingal C., Labrosse H., Farsi F., Dayde D. (2025). Development and validation of a food-related quality of life questionnaire (CANUT-QVA) for cancer patients. Clin. Nutr. Open Sci..

[19] Wądołowska L., Krusińska B. (2022). Procedura Opracowania Danych Żywieniowych z Kwestionariusza QEB. http://www.uwm.edu.pl/edu/lidiawadolowska.

[20] Cieślik B., Podbielska H. (2015). Przegląd wybranych kwestionariuszy oceny jakości życia. Inż. Biomed..

[21] Wołowicka L., Jaracz K., Wołowicka L. (2001). Polska wersja WHOQOL 100 i WHOQOL Bref. Jakość Życia W Naukach Medycznych.

[22] World Health Organization (1996). Quality of Life Group: WHOQOL-BREF Introduction. Administration and Scoring. Field Trial Version. https://www.who.int/publications/i/item/WHOQOL-BREF.

[23] Bani-Issa W. (2011). Evaluation of the health-related quality of life of Emirati people with diabetes: Integration of sociodemographic and disease-related variables. East. Mediterr. Health J..

[24] Schober P., Boer C., Schwarte L.A. (2018). Correlation Coefficients: Appropriate Use and Interpretation. Anesth. Analg..

[25] Islam N., Atreya A., Nepal S., Uddin K.J., Kaiser M.R., Menezes R.G., Lasrado S., Abdullah-Al-Noman M. (2023). Assessment of quality of life (QOL) in cancer patients attending oncology unit of a Teaching Hospital in Bangladesh. Cancer Rep..

[26] Lim L., Machingura A., Taye M., Pe M., Coens C., Martinelli F., Alanya A., Antunes S., Tu D., Basch E. (2025). Prognostic value of baseline EORTC QLQ-C30 scores for overall survival across 46 clinical trials covering 17 cancer types: A validation study. eClinicalMedicine.

[27] Wiese M.L., Schwarz L., von Rheinbaben S., Schmidt S., Blaurock M., Krönke J., Lerch M.M., Valentini L., Aghdassi A.A. (2026). Malnutrition in gastrointestinal cancer manifests before systemic therapy and is associated with fatigue and reduced physical quality of life. Oncologist.

[28] Abd Rashid A.A., Jan Mohamed H.J., Mitra A.K., Ashari L.S., Shahril M.R., Yeh L.Y., Ali R.A.R. (2025). Nutritional Association of Quality of Life Among Colorectal Cancer Survivors in Malaysia: A 6-Month Follow-Up Study. Int. J. Environ. Res. Public Health.

[29] Guo Z.Q., Yu J.M., Li W., Fu Z.M., Lin Y., Shi Y.Y., Hu W., Ba Y., Li S.Y., Li Z.N. (2020). Survey and analysis of the nutritional status in hospitalized patients with malignant gastric tumors and its influence on the quality of life. Support Care Cancer.

[30] Poulia K.A., Antoniadou D., Sarantis P., Karamouzis M.V. (2022). Pancreatic Cancer Prognosis, Malnutrition Risk, and Quality of Life: A Cross-Sectional Study. Nutrients.

[31] Lewandowska A., Rudzki G., Lewandowski T., Próchnicki M., Rudzki S., Laskowska B., Brudniak J. (2020). Quality of Life of Cancer Patients Treated with Chemotherapy. Int. J. Environ. Res. Public Health.

[32] Pękała M., Kozaka J. (2016). Quality of life of lung cancer patients. Psychoonkologia.

[33] Mziray M., Żuralska R., Postrożny D., Domagała P. (2014). The Feeling of Depression, Anxiety and Pain and Styles of Coping with Difficult Situations in Patients with Cancer. Przeds. Zarz..

[34] Pasek M., Urbański K., Suchocka L. (2013). Quality of life in advanced cervical cancer patients subjected to radiotherapy—A WHOQOL BREF questionnaire study. Psycho-Oncol..

[35] Ejupi V., Gori N., Gashi A.M. (2023). Quality of life in cancer patients—A systematic review. Ro Med. J..

[36] Rabin E.G., Heldt E., Hirakata V.N., Fleck M.P. (2008). Quality of life predictors in breast cancer women. Eur. J. Oncol. Nurs..

[37] Dąbrowska-Ilnicka J.W., Milska-Musa K.A. (2026). Social support and quality of life among oncology patients. Oncol. Clin. Pract..

[38] Bossi P., De Luca R., Ciani O., D’Angelo E., Caccialanza R. (2022). Malnutrition management in oncology: An expert view on controversial issues and future perspectives. Front. Oncol..

[39] Budai B.C., Martinekova P., Cai G., Dobszai D., Fekete L., Normann H.A., Németh J., Fazekas A., Szalai E.Á., Szentesi A. (2026). Risk of Malnutrition in Digestive System Cancers: A Systematic Review and Meta-Analysis. Cancers.

[40] Liu Z., She T., Mo S., Wei W., Guo Y., Liu Y., Zhang Y. (2026). Food and nutrition literacy and diet self-efficacy among patients with liver cancer: Latent profile analysis, associated factors, and an exploratory examination of gender differences. Front. Nutr..

[41] Miller S.M., Hudson S.V., Egleston B.L., Manne S., Buzaglo J.S., Devarajan K., Fleisher L., Millard J., Solarino N., Trinastic J. (2013). The relationships among knowledge, self-efficacy, preparedness, decisional conflict, and decisions to participate in a cancer clinical trial. Psycho-Oncol..

[42] Hanna L., Nguo K., Furness K., Porter J., Huggins C.E. (2022). Association between skeletal muscle mass and quality of life in adults with cancer: A systematic review and meta-analysis. J. Cachexia Sarcopenia Muscle.

[43] Ford K.L., Orsso C.E., Kiss N., Johnson S.B., Purcell S.A., Gagnon A., Laviano A., Prado C.M. (2022). Dietary Choices After a Cancer Diagnosis: A Narrative Review. Nutrition.

[44] Tan S.Y., Wong H.Y., Vardy J.L. (2021). Do cancer survivors change their diet after cancer diagnosis?. Support Care Cancer.

[45] Clotas C., Serral G., Vidal Garcia E., Puigpinós-Riera R., DAMA Cohort Group (2021). Dietary changes and food habits: Social and clinical determinants in a cohort of women diagnosed with breast cancer in Barcelona (DAMA cohort). Cancer Causes Control CCC.

[46] Krusińska B., Hawrysz I., Słowińska M.A., Wądołowska L., Biernacki M., Czerwińska A., Gołota J.J. (2017). Dietary patterns and breast or lung cancer risk: A pooled analysis of 2 case-control studies in north-eastern Poland. Adv. Clin. Exp. Med..

[47] Rim C.H., Ahn S.J., Kim J.H., Yoon W.S., Chun M., Yang D.S., Lee J.H., Kim K., Kong M., Kim S. (2019). Questionnaire study of the dietary habits of breast cancer survivors and their relationship to quality of life (KROG 14-09). Eur. J. Cancer Care.

[48] Aldossari A., Sremanakova J., Sowerbutts A.M., Jones D., Hann M., Burden S.T. (2023). Do people change their eating habits after a diagnosis of cancer? A systematic review. J. Hum. Nutr. Diet..

